# *N*′-(3-Eth­oxy-2-hy­droxy­benzyl­idene)-2-hy­droxy-3-methyl­benzohydrazide

**DOI:** 10.1107/S1600536812002127

**Published:** 2012-01-21

**Authors:** Zhao-Fu Zhu, Li-Juen Shao, Xi-Hai Shen

**Affiliations:** a Department of Chemistry, Hebei Normal University of Science and Technology, Qinhuangdao 066600, People’s Republic of China

## Abstract

The title compound, C_17_H_18_N_2_O_4_, crystallizes with two independent mol­ecules in the asymmetric unit. The two benzene rings in each mol­ecule make dihedral angles of 7.6 (3) and 3.9 (3)°. Intra­molecular O—H⋯N and O—H⋯O hydrogen bonds are present in each mol­ecule. In the crystal, N—H⋯O hydrogen bonds link the mol­ecules into chains propagating in [010]. The are also a number of C—H⋯O and π–π inter­actions present [centroid–centroid distances = 3.874 (4) and 3.904 (3) Å], that result in the formation of a three-dimensional network.

## Related literature

For the crystal structures of similar hydrazone compounds, see: Fun *et al.* (2011 ▶); Horkaew *et al.* (2011 ▶); Zhi *et al.* (2011 ▶); Huang & Wu (2010 ▶); Shen *et al.* (2012 ▶).
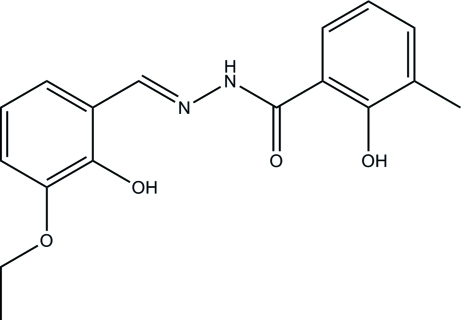


## Experimental

### 

#### Crystal data

C_17_H_18_N_2_O_4_
*M* *_r_* = 314.33
Monoclinic, 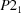

*a* = 7.7661 (17) Å
*b* = 22.148 (3) Å
*c* = 9.7002 (16) Å
β = 100.382 (2)°
*V* = 1641.1 (5) Å^3^
*Z* = 4
Mo *K*α radiation
μ = 0.09 mm^−1^
*T* = 298 K
0.20 × 0.20 × 0.18 mm

#### Data collection

Bruker SMART CCD area-detector diffractometer
Absorption correction: multi-scan (*SADABS*; Bruker, 2001 ▶) *T*_min_ = 0.982, *T*_max_ = 0.984
8061 measured reflections
3436 independent reflections
1592 reflections with *I* > 2σ(*I*)
*R*_int_ = 0.058

#### Refinement

*R*[*F* ^2^ > 2σ(*F* ^2^)] = 0.054
*wR*(*F* ^2^) = 0.113
*S* = 0.97
3436 reflections
431 parameters
4 restraints
H atoms treated by a mixture of independent and constrained refinement
Δρ_max_ = 0.13 e Å^−3^
Δρ_min_ = −0.15 e Å^−3^

### 

Data collection: *SMART* (Bruker, 2007 ▶); cell refinement: *SAINT* (Bruker, 2007 ▶); data reduction: *SAINT*; program(s) used to solve structure: *SHELXS97* (Sheldrick, 2008 ▶); program(s) used to refine structure: *SHELXL97* (Sheldrick, 2008 ▶); molecular graphics: *SHELXTL* (Sheldrick, 2008 ▶); software used to prepare material for publication: *SHELXTL*.

## Supplementary Material

Crystal structure: contains datablock(s) global, I. DOI: 10.1107/S1600536812002127/su2365sup1.cif

Structure factors: contains datablock(s) I. DOI: 10.1107/S1600536812002127/su2365Isup2.hkl

Supplementary material file. DOI: 10.1107/S1600536812002127/su2365Isup3.cml

Additional supplementary materials:  crystallographic information; 3D view; checkCIF report

## Figures and Tables

**Table 1:** Hydrogen-bond geometry (Å, °)

*D*—H⋯*A*	*D*—H	H⋯*A*	*D*⋯*A*	*D*—H⋯*A*
O1—H1⋯N1	0.82	1.84	2.558 (5)	145
O4—H4*B*⋯O3	0.82	1.83	2.549 (5)	145
O5—H5⋯N3	0.82	1.87	2.585 (6)	145
O8—H8⋯O6	0.85 (3)	1.75 (4)	2.536 (6)	152 (5)
N2—H2⋯O5^i^	0.90 (2)	2.22 (2)	3.035 (6)	150 (4)
N2—H2⋯O7^i^	0.90 (2)	2.52 (4)	3.218 (6)	134 (3)
N4—H4⋯O1	0.90 (4)	2.26 (3)	3.027 (5)	144 (5)
C7—H7⋯O5^i^	0.93	2.59	3.353 (7)	140
C14—H14⋯O5^i^	0.93	2.60	3.516 (7)	169
C24—H24⋯O1	0.93	2.45	3.261 (6)	145
C33—H33*A*⋯O6^ii^	0.97	2.58	3.420 (7)	145

Symmetry codes: (i) 
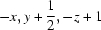
; (ii) 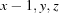.

**Fig. 1.**
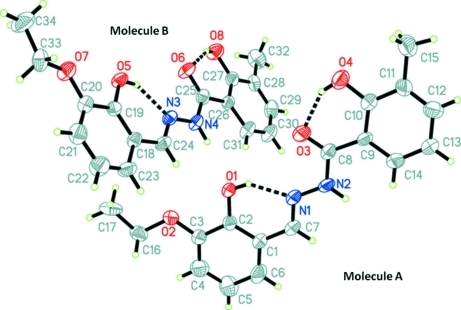
The molecular structure of the two independent molecules (A and B) of the title compound, with atom numbering and displacement ellipsoids drawn at the 30% probability level. The intramolecular O—H···O and O—H···N hydrogen bonds are drawn as dashed lines - see Table 1 for details.

**Fig. 2.**
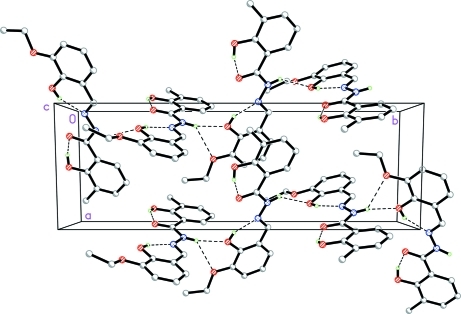
A partial view of the crystal packing of the title compound, viewed along the c axis. The O-H···O, O-H···N and N-H···O hydrogen bonds are drawn as dashed lines - see Table 1 for details.

## supplementary crystallographic information

### Comment

In the last few years, the crystal structures of a number of hydrazone compounds have been reported (Fun *et al.*, 2011; Horkaew *et al.*, 2011; Zhi *et al.*, 2011; Huang & Wu, 2010). However, compounds derived from 2-hydroxy-3-methylbenzohydrazide have seldom been reported. As an extension of our work on such compounds (Shen *et al.*, 2012), we report herein on the crystal structure of the title compound, prepared by condensing 3-ethoxy-2-hydroxybenzaldehyde and 2-hydroxy-3-methylbenzohydrazide in methanol.

The asymmetric unit of the title compound contains two independent molecules (A & B), Fig. 1. In both molecules there are intramoleculoar O-H···N and O-H···O hydrogen bonds (Table 1).

In molecule A the (C1—C6) and (C9—C14) benzene rings make a dihedral angle of 7.6 (3)°. In molecule B the (C18—C23) and (C26—C31) benzene rings make a dihedral angle of 3.9 (3)°. All the bond values are within normal ranges and are comparable with those in the similar compounds reported on by (Fun *et al.*, 2011; Horkaew *et al.*, 2011; Zhi *et al.*, 2011; Huang & Wu, 2010; Shen *et al.*, 2012).

In the crystal, there are intermolecular N—H···O hydrogen bonds linking the molecules to form -A-B-A-B- chains propagating along the b axis direction. The are a number of C-H···O interactions present (Table 1), and some π–π interactions involving symmetry related A/A molecules and neighbouring B/B molecules [Cg1—Cg2^i^ 3.874 (4) Å; symmetry code: (i) -*x*, *y*+172, -*z*+1; Cg3—Cg4^ii^ 3.904 (3) Å; symmetry code: (ii) *x*-1, *y*, *z*; where Cg1, Cg2, Cg3, and Cg4 are the centroids of the (C1-C6), (C9-C14), (C18-C23) and (C26-C31) benzene rings, respectively]. The sum of these interactions results in the formation of a three-dimensional network.

### Experimental

3-Ethoxy-2-hydroxybenzaldehyde (166.2 mg, 1.0 mmol) and 2-hydroxy-3-methylbenzohydrazide (166.2 mg, 1.0 mmol) were mixed in methanol (60 ml), and refluxed for 30 min, then cooled to room temperature, yielding colourless solution. Colourless block-like crystals of the title compound were formed when the solution was evaporated in air for several days.

### Refinement

The amino H atoms were located in a difference Fourier map and were refined with the N—H distances restrained to 0.90 (1) Å. The (O8) hydroxyl H atom was also located in a difference Fourier map and was freely refined with *U*_iso_(H8) = *U*_eq_(O8). The remaining H atoms were placed in idealized positions and constrained to ride on their parent atoms: O-H = 0.82 Å, C-H = 0.93, 0.97 and 0.96 Å for CH, CH_2_ and CH_3_ H-atoms, respectively, with U_iso_(H) = k × U_eq_(C), where k = 1.5 for OH and CH_3_ H-atoms, and k = 1.2 for all other H-atoms. In the final cycles of refinement, in the absence of significant anomalous scattering effects, the Friedel pairs were merged and Δf " set to zero.

### Crystal data

**Table:**

C_17_H_18_N_2_O_4_	*F*(000) = 664
*M**_r_* = 314.33	*D*_x_ = 1.272 Mg m^−^^3^
Monoclinic, *P*2_1_	Mo *K*α radiation, λ = 0.71073 Å
Hall symbol: P 2yb	Cell parameters from 756 reflections
*a* = 7.7661 (17) Å	θ = 2.3–24.1°
*b* = 22.148 (3) Å	µ = 0.09 mm^−^^1^
*c* = 9.7002 (16) Å	*T* = 298 K
β = 100.382 (2)°	Block, colourless
*V* = 1641.1 (5) Å^3^	0.20 × 0.20 × 0.18 mm
*Z* = 4	

### Data collection

**Table:**

Bruker SMART CCD area-detector diffractometer	3436 independent reflections
Radiation source: fine-focus sealed tube	1592 reflections with *I* > 2σ(*I*)
graphite	*R*_int_ = 0.058
ω scans	θ_max_ = 26.5°, θ_min_ = 2.1°
Absorption correction: multi-scan (*SADABS*; Bruker, 2001)	*h* = −7→9
*T*_min_ = 0.982, *T*_max_ = 0.984	*k* = −26→27
8061 measured reflections	*l* = −11→12

### Refinement

**Table:**

Refinement on *F*^2^	Primary atom site location: structure-invariant direct methods
Least-squares matrix: full	Secondary atom site location: difference Fourier map
*R*[*F*^2^ > 2σ(*F*^2^)] = 0.054	Hydrogen site location: inferred from neighbouring sites
*wR*(*F*^2^) = 0.113	H atoms treated by a mixture of independent and constrained refinement
*S* = 0.97	*w* = 1/[σ^2^(*F*_o_^2^) + (0.033*P*)^2^] where *P* = (*F*_o_^2^ + 2*F*_c_^2^)/3
3436 reflections	(Δ/σ)_max_ < 0.001
431 parameters	Δρ_max_ = 0.13 e Å^−^^3^
4 restraints	Δρ_min_ = −0.15 e Å^−^^3^

### Special details

**Table:**

Geometry. Bond distances, angles etc. have been calculated using the rounded fractional coordinates. All su's are estimated from the variances of the (full) variance-covariance matrix. The cell esds are taken into account in the estimation of distances, angles and torsion angles
Refinement. Refinement of *F*^2^ against ALL reflections. The weighted *R*-factor *wR* and goodness of fit *S* are based on *F*^2^, conventional *R*-factors *R* are based on *F*, with *F* set to zero for negative *F*^2^. The threshold expression of *F*^2^ > σ(*F*^2^) is used only for calculating *R*-factors(gt) *etc*. and is not relevant to the choice of reflections for refinement. *R*-factors based on *F*^2^ are statistically about twice as large as those based on *F*, and *R*- factors based on ALL data will be even larger.

### Fractional atomic coordinates and isotropic or equivalent isotropic displacement parameters (Å^2^)

**Table:**

	*x*	*y*	*z*	*U*_iso_*/*U*_eq_	
O1	0.1824 (5)	0.21340 (14)	0.6459 (3)	0.0614 (14)	
O2	0.2554 (5)	0.17087 (16)	0.9028 (4)	0.0698 (16)	
O3	0.0043 (6)	0.24281 (18)	0.2650 (4)	0.0890 (19)	
O4	−0.1208 (6)	0.23100 (17)	0.0048 (4)	0.0893 (17)	
N1	0.1655 (6)	0.3042 (2)	0.4810 (5)	0.0631 (17)	
N2	0.1167 (6)	0.3313 (2)	0.3504 (5)	0.0658 (19)	
C1	0.2983 (7)	0.3115 (3)	0.7185 (6)	0.063 (2)	
C2	0.2635 (6)	0.2517 (2)	0.7481 (5)	0.057 (2)	
C3	0.3042 (7)	0.2287 (3)	0.8847 (6)	0.065 (2)	
C4	0.3899 (9)	0.2654 (4)	0.9877 (7)	0.104 (3)	
C5	0.4308 (10)	0.3244 (4)	0.9603 (8)	0.126 (4)	
C6	0.3823 (9)	0.3481 (3)	0.8288 (8)	0.099 (3)	
C7	0.2473 (7)	0.3374 (3)	0.5801 (6)	0.068 (3)	
C8	0.0341 (8)	0.2960 (3)	0.2444 (6)	0.064 (2)	
C9	−0.0216 (7)	0.3263 (2)	0.1074 (6)	0.057 (2)	
C10	−0.0924 (7)	0.2907 (3)	−0.0083 (6)	0.069 (2)	
C11	−0.1371 (8)	0.3159 (3)	−0.1414 (7)	0.080 (3)	
C12	−0.1147 (9)	0.3763 (4)	−0.1557 (7)	0.097 (3)	
C13	−0.0494 (9)	0.4132 (3)	−0.0452 (7)	0.094 (3)	
C14	−0.0006 (8)	0.3884 (3)	0.0874 (6)	0.081 (3)	
C15	−0.2122 (9)	0.2749 (3)	−0.2636 (7)	0.115 (3)	
C16	0.2750 (8)	0.1487 (3)	1.0453 (6)	0.081 (3)	
C17	0.1917 (9)	0.0870 (3)	1.0394 (6)	0.101 (3)	
O5	−0.1527 (4)	−0.03370 (15)	0.5987 (4)	0.0601 (16)	
O6	0.2365 (5)	0.00000 (16)	0.4276 (4)	0.0735 (17)	
O7	−0.4309 (5)	−0.07716 (17)	0.6760 (4)	0.0754 (17)	
O8	0.5012 (5)	−0.01235 (19)	0.3106 (4)	0.0801 (17)	
N3	0.0562 (6)	0.05665 (19)	0.5918 (4)	0.0544 (17)	
N4	0.1951 (6)	0.0840 (2)	0.5477 (5)	0.0597 (17)	
C18	−0.1801 (7)	0.0613 (2)	0.7159 (5)	0.053 (2)	
C19	−0.2382 (7)	0.0038 (2)	0.6776 (5)	0.0515 (19)	
C20	−0.3885 (7)	−0.0194 (3)	0.7188 (6)	0.061 (2)	
C21	−0.4786 (8)	0.0161 (3)	0.7980 (6)	0.082 (3)	
C22	−0.4209 (9)	0.0736 (3)	0.8346 (7)	0.089 (3)	
C23	−0.2746 (8)	0.0965 (3)	0.7968 (6)	0.075 (3)	
C24	−0.0276 (7)	0.0874 (2)	0.6696 (6)	0.060 (2)	
C25	0.2855 (7)	0.0518 (3)	0.4634 (6)	0.060 (2)	
C26	0.4379 (7)	0.0804 (2)	0.4208 (5)	0.055 (2)	
C27	0.5402 (8)	0.0456 (3)	0.3454 (6)	0.061 (2)	
C28	0.6896 (8)	0.0694 (3)	0.3020 (6)	0.071 (3)	
C29	0.7313 (8)	0.1279 (3)	0.3357 (7)	0.082 (3)	
C30	0.6338 (9)	0.1639 (3)	0.4091 (7)	0.087 (3)	
C31	0.4889 (8)	0.1399 (3)	0.4511 (6)	0.077 (3)	
C32	0.7986 (9)	0.0305 (4)	0.2240 (7)	0.106 (3)	
C33	−0.6007 (7)	−0.0994 (3)	0.6885 (6)	0.080 (3)	
C34	−0.6145 (10)	−0.1627 (3)	0.6347 (8)	0.124 (4)	
H1	0.16610	0.23080	0.57010	0.0920*	
H2	0.147 (6)	0.3700 (9)	0.338 (5)	0.0800*	
H4A	0.42150	0.25040	1.07830	0.1240*	
H4B	−0.10510	0.22200	0.08810	0.1340*	
H5A	0.49160	0.34820	1.03180	0.1520*	
H6	0.40510	0.38840	0.81230	0.1180*	
H7	0.27330	0.37740	0.56320	0.0810*	
H12	−0.14490	0.39330	−0.24440	0.1170*	
H13	−0.03800	0.45450	−0.05900	0.1120*	
H14	0.04590	0.41280	0.16290	0.0980*	
H15A	−0.24110	0.29850	−0.34760	0.1720*	
H15B	−0.31570	0.25540	−0.24440	0.1720*	
H15C	−0.12690	0.24490	−0.27580	0.1720*	
H16A	0.21790	0.17580	1.10140	0.0970*	
H16B	0.39800	0.14610	1.08710	0.0970*	
H17A	0.06800	0.09060	1.00610	0.1510*	
H17B	0.21200	0.06940	1.13140	0.1510*	
H17C	0.24200	0.06160	0.97680	0.1510*	
H4	0.243 (7)	0.1196 (13)	0.577 (5)	0.0800*	
H5	−0.07000	−0.01560	0.57610	0.0900*	
H8	0.397 (3)	−0.015 (3)	0.327 (6)	0.0800*	
H21	−0.57850	0.00130	0.82670	0.0980*	
H22	−0.48410	0.09740	0.88680	0.1080*	
H23	−0.23710	0.13530	0.82430	0.0910*	
H24	0.00870	0.12640	0.69670	0.0720*	
H29	0.82990	0.14440	0.30810	0.0990*	
H30	0.66600	0.20380	0.42960	0.1050*	
H31	0.42330	0.16390	0.50080	0.0920*	
H32A	0.73200	0.02030	0.13370	0.1580*	
H32B	0.83150	−0.00580	0.27630	0.1580*	
H32C	0.90200	0.05220	0.21210	0.1580*	
H33A	−0.69100	−0.07450	0.63430	0.0950*	
H33B	−0.61510	−0.09850	0.78580	0.0950*	
H34A	−0.60150	−0.16290	0.53820	0.1870*	
H34B	−0.72690	−0.17890	0.64270	0.1870*	
H34C	−0.52410	−0.18680	0.68870	0.1870*	

### Atomic displacement parameters (Å^2^)

**Table:**

	*U*^11^	*U*^22^	*U*^33^	*U*^12^	*U*^13^	*U*^23^
O1	0.084 (3)	0.041 (2)	0.058 (2)	−0.008 (2)	0.010 (2)	−0.0046 (18)
O2	0.089 (3)	0.061 (3)	0.060 (2)	−0.002 (2)	0.0147 (19)	0.006 (2)
O3	0.132 (4)	0.049 (3)	0.084 (3)	−0.022 (3)	0.014 (2)	0.005 (2)
O4	0.105 (3)	0.057 (3)	0.100 (3)	−0.002 (3)	0.003 (3)	−0.009 (2)
N1	0.076 (3)	0.040 (3)	0.073 (3)	−0.006 (3)	0.013 (3)	0.000 (3)
N2	0.084 (4)	0.037 (3)	0.075 (3)	−0.006 (3)	0.011 (3)	0.009 (3)
C1	0.067 (4)	0.048 (4)	0.072 (4)	0.001 (3)	0.011 (3)	−0.002 (3)
C2	0.059 (4)	0.054 (4)	0.056 (4)	−0.003 (3)	0.007 (3)	−0.003 (3)
C3	0.073 (4)	0.058 (4)	0.062 (4)	−0.001 (3)	0.010 (3)	−0.002 (3)
C4	0.141 (7)	0.085 (6)	0.075 (5)	−0.024 (5)	−0.006 (4)	−0.002 (4)
C5	0.172 (8)	0.113 (7)	0.074 (5)	−0.030 (6)	−0.030 (5)	−0.025 (5)
C6	0.126 (6)	0.058 (4)	0.104 (5)	−0.023 (4)	−0.002 (5)	−0.022 (4)
C7	0.075 (4)	0.043 (4)	0.086 (5)	−0.001 (3)	0.016 (3)	0.003 (3)
C8	0.070 (4)	0.047 (4)	0.077 (4)	−0.001 (3)	0.018 (3)	−0.002 (3)
C9	0.067 (4)	0.039 (3)	0.065 (4)	−0.001 (3)	0.010 (3)	0.009 (3)
C10	0.071 (4)	0.057 (4)	0.078 (4)	0.007 (3)	0.014 (3)	−0.001 (4)
C11	0.093 (5)	0.078 (5)	0.066 (4)	0.019 (4)	0.005 (4)	−0.003 (4)
C12	0.126 (6)	0.094 (6)	0.075 (5)	0.024 (5)	0.027 (4)	0.016 (4)
C13	0.145 (7)	0.053 (4)	0.082 (5)	0.003 (4)	0.019 (5)	0.007 (4)
C14	0.108 (5)	0.060 (4)	0.074 (4)	−0.012 (4)	0.012 (4)	0.012 (3)
C15	0.129 (6)	0.119 (6)	0.086 (5)	0.026 (5)	−0.007 (4)	−0.018 (5)
C16	0.081 (4)	0.102 (5)	0.060 (4)	0.024 (4)	0.011 (3)	0.017 (4)
C17	0.106 (6)	0.106 (6)	0.092 (5)	−0.007 (5)	0.021 (4)	0.049 (5)
O5	0.064 (3)	0.041 (2)	0.079 (3)	−0.0014 (18)	0.023 (2)	−0.010 (2)
O6	0.072 (3)	0.045 (3)	0.108 (3)	−0.011 (2)	0.028 (2)	−0.013 (2)
O7	0.066 (3)	0.062 (3)	0.101 (3)	−0.011 (2)	0.023 (2)	−0.005 (2)
O8	0.078 (3)	0.064 (3)	0.104 (3)	0.001 (3)	0.032 (3)	−0.012 (2)
N3	0.047 (3)	0.048 (3)	0.066 (3)	−0.007 (2)	0.004 (2)	0.000 (2)
N4	0.060 (3)	0.040 (3)	0.081 (3)	−0.006 (3)	0.018 (3)	0.003 (3)
C18	0.053 (4)	0.042 (3)	0.066 (4)	0.005 (3)	0.014 (3)	0.003 (3)
C19	0.056 (4)	0.045 (3)	0.056 (3)	0.012 (3)	0.017 (3)	0.001 (3)
C20	0.059 (4)	0.055 (4)	0.070 (4)	−0.001 (3)	0.013 (3)	0.005 (3)
C21	0.077 (5)	0.078 (5)	0.100 (5)	0.009 (4)	0.041 (4)	0.006 (4)
C22	0.091 (5)	0.074 (5)	0.116 (6)	0.008 (4)	0.053 (4)	−0.011 (4)
C23	0.091 (5)	0.053 (4)	0.085 (4)	−0.002 (4)	0.025 (4)	−0.019 (3)
C24	0.065 (4)	0.038 (3)	0.074 (4)	0.005 (3)	0.004 (3)	−0.002 (3)
C25	0.055 (4)	0.057 (4)	0.065 (4)	0.003 (3)	0.007 (3)	0.008 (3)
C26	0.060 (4)	0.045 (4)	0.059 (3)	0.000 (3)	0.009 (3)	0.006 (3)
C27	0.060 (4)	0.061 (4)	0.058 (4)	−0.004 (3)	0.001 (3)	0.007 (3)
C28	0.078 (5)	0.074 (5)	0.060 (4)	0.004 (4)	0.012 (3)	0.020 (3)
C29	0.064 (5)	0.091 (6)	0.095 (5)	−0.008 (4)	0.023 (4)	0.023 (4)
C30	0.085 (5)	0.061 (4)	0.117 (5)	−0.018 (4)	0.021 (4)	0.007 (4)
C31	0.073 (4)	0.051 (4)	0.108 (5)	−0.009 (4)	0.019 (4)	0.005 (3)
C32	0.083 (5)	0.136 (7)	0.106 (5)	0.007 (5)	0.040 (4)	0.002 (5)
C33	0.065 (4)	0.086 (5)	0.090 (4)	−0.008 (4)	0.021 (3)	0.024 (4)
C34	0.123 (7)	0.105 (7)	0.154 (7)	−0.064 (5)	0.049 (5)	−0.040 (5)

### Geometric parameters (Å, °)

**Table:**

O1—C2	1.369 (6)	C12—H12	0.9300
O2—C3	1.356 (7)	C13—H13	0.9300
O2—C16	1.449 (7)	C14—H14	0.9300
O3—C8	1.224 (8)	C15—H15C	0.9600
O4—C10	1.350 (8)	C15—H15A	0.9600
O1—H1	0.8200	C15—H15B	0.9600
O4—H4B	0.8200	C16—H16A	0.9700
O5—C19	1.378 (6)	C16—H16B	0.9700
O6—C25	1.239 (7)	C17—H17B	0.9600
O7—C20	1.367 (8)	C17—H17A	0.9600
O7—C33	1.433 (7)	C17—H17C	0.9600
O8—C27	1.348 (8)	C18—C19	1.380 (6)
O5—H5	0.8200	C18—C24	1.460 (8)
O8—H8	0.85 (3)	C18—C23	1.404 (8)
N1—N2	1.391 (7)	C19—C20	1.398 (8)
N1—C7	1.284 (8)	C20—C21	1.375 (9)
N2—C8	1.357 (8)	C21—C22	1.375 (9)
N2—H2	0.90 (2)	C22—C23	1.354 (9)
N3—N4	1.371 (7)	C25—C26	1.466 (8)
N3—C24	1.278 (7)	C26—C27	1.403 (8)
N4—C25	1.370 (8)	C26—C31	1.392 (8)
N4—H4	0.90 (4)	C27—C28	1.406 (9)
C1—C2	1.392 (8)	C28—C32	1.504 (10)
C1—C6	1.406 (9)	C28—C29	1.361 (9)
C1—C7	1.448 (8)	C29—C30	1.382 (9)
C2—C3	1.402 (8)	C30—C31	1.371 (9)
C3—C4	1.365 (10)	C33—C34	1.493 (9)
C4—C5	1.382 (12)	C21—H21	0.9300
C5—C6	1.368 (11)	C22—H22	0.9300
C8—C9	1.482 (8)	C23—H23	0.9300
C9—C14	1.403 (8)	C24—H24	0.9300
C9—C10	1.400 (8)	C29—H29	0.9300
C10—C11	1.392 (9)	C30—H30	0.9300
C11—C12	1.359 (11)	C31—H31	0.9300
C11—C15	1.523 (9)	C32—H32A	0.9600
C12—C13	1.370 (10)	C32—H32B	0.9600
C13—C14	1.387 (9)	C32—H32C	0.9600
C16—C17	1.509 (9)	C33—H33A	0.9700
C4—H4A	0.9300	C33—H33B	0.9700
C5—H5A	0.9300	C34—H34A	0.9600
C6—H6	0.9300	C34—H34B	0.9600
C7—H7	0.9300	C34—H34C	0.9600
			
C3—O2—C16	117.4 (4)	H16A—C16—H16B	109.00
C2—O1—H1	110.00	H17B—C17—H17C	109.00
C10—O4—H4B	109.00	H17A—C17—H17C	109.00
C20—O7—C33	118.1 (4)	C16—C17—H17B	110.00
C19—O5—H5	109.00	C16—C17—H17A	109.00
C27—O8—H8	101 (4)	H17A—C17—H17B	109.00
N2—N1—C7	116.8 (5)	C16—C17—H17C	110.00
N1—N2—C8	117.1 (5)	C19—C18—C24	121.8 (5)
N1—N2—H2	120 (3)	C23—C18—C24	119.2 (5)
C8—N2—H2	123 (3)	C19—C18—C23	119.0 (5)
N4—N3—C24	117.5 (4)	O5—C19—C20	116.6 (4)
N3—N4—C25	118.2 (4)	C18—C19—C20	120.8 (5)
N3—N4—H4	127 (3)	O5—C19—C18	122.6 (5)
C25—N4—H4	115 (3)	O7—C20—C19	115.2 (5)
C6—C1—C7	119.3 (6)	O7—C20—C21	125.9 (5)
C2—C1—C6	118.2 (5)	C19—C20—C21	119.0 (6)
C2—C1—C7	122.5 (5)	C20—C21—C22	119.9 (6)
O1—C2—C1	121.4 (4)	C21—C22—C23	121.8 (6)
O1—C2—C3	117.1 (4)	C18—C23—C22	119.5 (6)
C1—C2—C3	121.5 (5)	N3—C24—C18	119.9 (4)
O2—C3—C2	116.4 (5)	O6—C25—N4	119.0 (5)
C2—C3—C4	118.2 (6)	O6—C25—C26	123.0 (5)
O2—C3—C4	125.4 (6)	N4—C25—C26	118.0 (5)
C3—C4—C5	121.4 (7)	C27—C26—C31	117.6 (5)
C4—C5—C6	120.5 (7)	C25—C26—C27	118.1 (5)
C1—C6—C5	120.0 (7)	C25—C26—C31	124.3 (5)
N1—C7—C1	118.9 (6)	O8—C27—C28	116.3 (5)
N2—C8—C9	116.1 (5)	C26—C27—C28	121.7 (6)
O3—C8—N2	120.5 (5)	O8—C27—C26	122.1 (5)
O3—C8—C9	123.3 (5)	C27—C28—C29	117.3 (6)
C8—C9—C10	118.2 (5)	C27—C28—C32	120.4 (6)
C8—C9—C14	123.1 (5)	C29—C28—C32	122.4 (6)
C10—C9—C14	118.7 (5)	C28—C29—C30	123.0 (6)
C9—C10—C11	120.9 (6)	C29—C30—C31	119.0 (6)
O4—C10—C9	121.5 (5)	C26—C31—C30	121.5 (6)
O4—C10—C11	117.5 (5)	O7—C33—C34	107.6 (5)
C12—C11—C15	123.1 (6)	C20—C21—H21	120.00
C10—C11—C15	118.6 (6)	C22—C21—H21	120.00
C10—C11—C12	118.3 (6)	C21—C22—H22	119.00
C11—C12—C13	122.9 (7)	C23—C22—H22	119.00
C12—C13—C14	119.3 (6)	C18—C23—H23	120.00
C9—C14—C13	119.8 (6)	C22—C23—H23	120.00
O2—C16—C17	107.5 (5)	N3—C24—H24	120.00
C3—C4—H4A	119.00	C18—C24—H24	120.00
C5—C4—H4A	119.00	C28—C29—H29	119.00
C4—C5—H5A	120.00	C30—C29—H29	119.00
C6—C5—H5A	120.00	C29—C30—H30	120.00
C1—C6—H6	120.00	C31—C30—H30	121.00
C5—C6—H6	120.00	C26—C31—H31	119.00
N1—C7—H7	121.00	C30—C31—H31	119.00
C1—C7—H7	121.00	C28—C32—H32A	110.00
C11—C12—H12	119.00	C28—C32—H32B	110.00
C13—C12—H12	119.00	C28—C32—H32C	109.00
C14—C13—H13	120.00	H32A—C32—H32B	109.00
C12—C13—H13	120.00	H32A—C32—H32C	109.00
C13—C14—H14	120.00	H32B—C32—H32C	109.00
C9—C14—H14	120.00	O7—C33—H33A	110.00
C11—C15—H15B	110.00	O7—C33—H33B	110.00
H15A—C15—H15B	110.00	C34—C33—H33A	110.00
C11—C15—H15A	110.00	C34—C33—H33B	110.00
H15B—C15—H15C	109.00	H33A—C33—H33B	108.00
H15A—C15—H15C	109.00	C33—C34—H34A	109.00
C11—C15—H15C	109.00	C33—C34—H34B	109.00
C17—C16—H16B	110.00	C33—C34—H34C	109.00
C17—C16—H16A	110.00	H34A—C34—H34B	110.00
O2—C16—H16A	110.00	H34A—C34—H34C	110.00
O2—C16—H16B	110.00	H34B—C34—H34C	110.00
			
C16—O2—C3—C2	−172.1 (5)	O4—C10—C11—C15	−0.9 (8)
C16—O2—C3—C4	8.5 (8)	C9—C10—C11—C12	−1.7 (9)
C3—O2—C16—C17	172.9 (5)	C9—C10—C11—C15	179.5 (6)
C33—O7—C20—C19	−167.2 (5)	C10—C11—C12—C13	0.4 (10)
C33—O7—C20—C21	13.5 (8)	C15—C11—C12—C13	179.1 (7)
C20—O7—C33—C34	179.8 (5)	C11—C12—C13—C14	1.1 (11)
C7—N1—N2—C8	178.4 (5)	C12—C13—C14—C9	−1.2 (10)
N2—N1—C7—C1	179.0 (5)	C23—C18—C19—O5	−179.8 (5)
N1—N2—C8—C9	178.1 (5)	C23—C18—C19—C20	−0.2 (8)
N1—N2—C8—O3	0.3 (8)	C24—C18—C19—O5	2.9 (8)
N4—N3—C24—C18	178.0 (4)	C24—C18—C19—C20	−177.5 (5)
C24—N3—N4—C25	−179.5 (5)	C19—C18—C23—C22	−0.5 (8)
N3—N4—C25—O6	1.6 (8)	C24—C18—C23—C22	176.9 (6)
N3—N4—C25—C26	−177.8 (4)	C19—C18—C24—N3	−0.2 (8)
C6—C1—C2—O1	−179.9 (5)	C23—C18—C24—N3	−177.6 (5)
C6—C1—C2—C3	2.5 (8)	O5—C19—C20—O7	0.5 (7)
C7—C1—C2—O1	1.8 (8)	O5—C19—C20—C21	179.8 (5)
C2—C1—C6—C5	1.4 (10)	C18—C19—C20—O7	−179.1 (5)
C7—C1—C6—C5	179.8 (6)	C18—C19—C20—C21	0.2 (8)
C7—C1—C2—C3	−175.9 (5)	O7—C20—C21—C22	179.6 (6)
C6—C1—C7—N1	−177.7 (6)	C19—C20—C21—C22	0.4 (9)
C2—C1—C7—N1	0.6 (8)	C20—C21—C22—C23	−1.1 (10)
C1—C2—C3—C4	−4.2 (8)	C21—C22—C23—C18	1.2 (10)
C1—C2—C3—O2	176.3 (5)	O6—C25—C26—C27	−5.2 (8)
O1—C2—C3—O2	−1.5 (7)	O6—C25—C26—C31	175.3 (5)
O1—C2—C3—C4	178.0 (5)	N4—C25—C26—C27	174.2 (5)
O2—C3—C4—C5	−178.4 (6)	N4—C25—C26—C31	−5.3 (8)
C2—C3—C4—C5	2.2 (10)	C25—C26—C27—O8	0.7 (8)
C3—C4—C5—C6	1.6 (12)	C25—C26—C27—C28	−179.3 (5)
C4—C5—C6—C1	−3.4 (11)	C31—C26—C27—O8	−179.8 (5)
O3—C8—C9—C10	−8.2 (9)	C31—C26—C27—C28	0.3 (8)
O3—C8—C9—C14	174.3 (6)	C25—C26—C31—C30	179.5 (6)
N2—C8—C9—C10	174.1 (5)	C27—C26—C31—C30	0.0 (8)
N2—C8—C9—C14	−3.4 (9)	O8—C27—C28—C29	179.9 (5)
C10—C9—C14—C13	−0.1 (9)	O8—C27—C28—C32	−1.3 (8)
C8—C9—C10—O4	4.4 (8)	C26—C27—C28—C29	−0.2 (9)
C8—C9—C10—C11	−176.0 (5)	C26—C27—C28—C32	178.7 (5)
C14—C9—C10—O4	−178.0 (5)	C27—C28—C29—C30	−0.2 (10)
C14—C9—C10—C11	1.6 (9)	C32—C28—C29—C30	−179.0 (6)
C8—C9—C14—C13	177.4 (6)	C28—C29—C30—C31	0.4 (10)
O4—C10—C11—C12	177.9 (6)	C29—C30—C31—C26	−0.3 (9)

### Hydrogen-bond geometry (Å, °)

**Table:**

*D*—H···*A*	*D*—H	H···*A*	*D*···*A*	*D*—H···*A*
O1—H1···N1	0.82	1.84	2.558 (5)	145
O4—H4B···O3	0.82	1.83	2.549 (5)	145
O5—H5···N3	0.82	1.87	2.585 (6)	145
O8—H8···O6	0.85 (3)	1.75 (4)	2.536 (6)	152 (5)
N2—H2···O5^i^	0.90 (2)	2.22 (2)	3.035 (6)	150 (4)
N2—H2···O7^i^	0.90 (2)	2.52 (4)	3.218 (6)	134 (3)
N4—H4···O1	0.90 (4)	2.26 (3)	3.027 (5)	144 (5)
C7—H7···O5^i^	0.93	2.59	3.353 (7)	140
C14—H14···O5^i^	0.93	2.60	3.516 (7)	169
C24—H24···O1	0.93	2.45	3.261 (6)	145
C33—H33A···O6^ii^	0.97	2.58	3.420 (7)	145

Symmetry codes: (i) −*x*, *y*+1/2, −*z*+1; (ii) *x*−1, *y*, *z*.
